# Applications of Medium-Chain Triglycerides in Foods

**DOI:** 10.3389/fnut.2022.802805

**Published:** 2022-06-02

**Authors:** Shinji Watanabe, Shougo Tsujino

**Affiliations:** Central Research Laboratory, The Nisshin OilliO Group, Ltd., Tokyo, Japan

**Keywords:** medium-chain triglycerides, medium-chain fatty acids, malnutrition, frailty, obesity, dementia

## Abstract

In the 1950s, the production of processed fats and oils from coconut oil was popular in the United States. It became necessary to find uses for the medium-chain fatty acids (MCFAs) that were byproducts of the process, and a production method for medium-chain triglycerides (MCTs) was established. At the time of this development, its use as a non-fattening fat was being studied. In the early days MCFAs included fatty acids ranging from hexanoic acid (C6:0) to dodecanoic acid (C12:0), but today their compositions vary among manufacturers and there seems to be no clear definition. MCFAs are more polar than long-chain fatty acids (LCFAs) because of their shorter chain length, and their hydrolysis and absorption properties differ greatly. These differences in physical properties have led, since the 1960s, to the use of MCTs to improve various lipid absorption disorders and malnutrition. More than half a century has passed since MCTs were first used in the medical field. It has been reported that they not only have properties as an energy source, but also have various physiological effects, such as effects on fat and protein metabolism. The enhancement of fat oxidation through ingestion of MCTs has led to interest in the study of body fat reduction and improvement of endurance during exercise. Recently, MCTs have also been shown to promote protein anabolism and inhibit catabolism, and applied research has been conducted into the prevention of frailty in the elderly. In addition, a relatively large ingestion of MCTs can be partially converted into ketone bodies, which can be used as a component of “ketone diets” in the dietary treatment of patients with intractable epilepsy, or in the nutritional support of terminally ill cancer patients. The possibility of improving cognitive function in dementia patients and mild cognitive impairment is also being studied. Obesity due to over-nutrition and lack of exercise, and frailty due to under-nutrition and aging, are major health issues in today's society. MCTs have been studied in relation to these concerns. In this paper we will introduce the results of applied research into the use of MCTs by healthy subjects.

## Introduction

**Figure 1:**
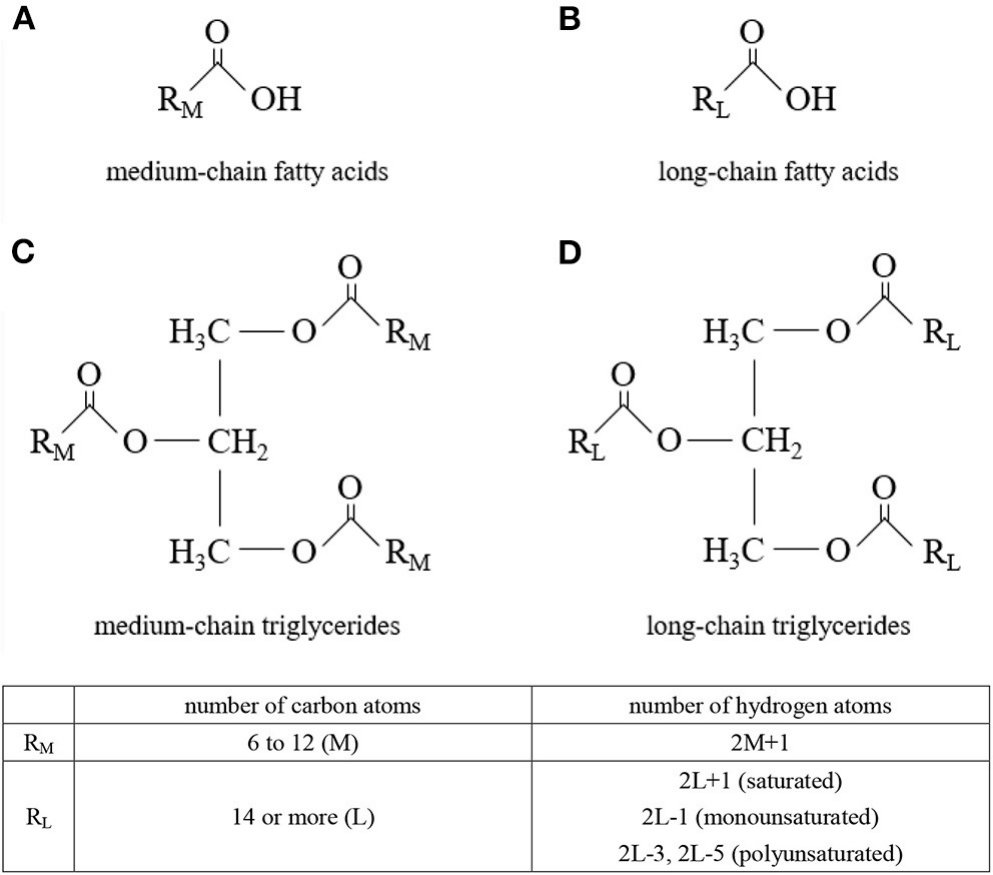
Molecular structures. **(A)** medium-chain fatty acids, **(B)** long-chain fatty acids, **(C)** medium-chain triglycerides, and **(D)** long-chain triglycerides.

Medium-chain triglycerides (MCTs) are composed of medium-chain fatty acids (MCFAs) (Figure 1). MCTs were developed as byproducts of coconut oil production in the 1950s, and research into their applications began. Since then, they have been used in a wide range of food and non-food applications. Although MCFAs are classified as saturated fatty acids, their nutritional, physiological, and physicochemical characteristics differ from those of so-called long-chain saturated fatty acids.

MCTs have been treated for decades since their development, mainly in medical applications for patients. However, in recent years, many studies on healthy subjects have been reported. Various social issues that reduce wellbeing have become apparent, such as the increasing number of obese young and middle-aged people, and frailty and cognitive decline among the elderly. Properly incorporating MCTs as food into our daily lives may help us lead healthier lives. We aim to explore the potential of MCTs as a solution to these issues and introduce the nutritional characteristics of MCTs, focusing on reports of clinical trials in healthy subjects.

## MCFAs and MCTs

The industrial production method of MCTs was developed by Babayan et al. at Drew Chemical in the 1950s, starting with the establishment of production and the development of applications for unused fatty acids with low melting points (C8:0 and C10:0) in coconut oil. Thus, most MCFAs in commercially available MCTs are composed of C8:0 and C10:0, with very little C12:0 (1). The composition of commercial MCTs products remains unchanged today, with most of them consisting of only C8:0 and C10:0. Mr. Drew of Drew Chemical is said to have instructed Mr. Babayan to use the unused fatty acids (C8:0 and C10:0) to produce “fatless fat” (2). Since then, MCTs have become available for experimental use. Kaunitz et al. at Columbia University confirmed in experiments on pigs that MCTs, which have twice the calories of lard, do not cause weight gain and are safe lipids (3). Subsequent human studies have confirmed that they are useful and safe for long-term use in patients with lipid absorption disorders (2), and then research into MCTs applications began.

There are various opinions on the definition of MCFAs, but in the broadest sense, the range from hexanoic acid (C6:0) to dodecanoic acid (C12:0) are considered to be MCFAs (4). In many cases (5), octanoic acid (C8:0) and decanoic acid (C10:0) are treated as MCFAs from the viewpoints of nutritional physiology, nutritional pharmacology, and physical chemistry, but there are no clear definitions. The fatty acids in coconut oil contain a total of about 60% of C8:0 to C12:0, but only about 10% of C8:0 and C10:0, and many long-chain saturated fatty acids (Table 1). Coconut oil has a significantly different composition of fatty acids than MCTs.

**Table 1:** Fatty acid composition [Modified from Edem (6)].

**Fatty acid**	**Red palm**	**Palm kernel**	**Coconut**
C6:0	–	0.2	0.5
C8:0	–	3.3	8.0
C10:0	–	3.5	6.4
C12:0	0.2	47.8	48.5
C14:0	1.1	16.3	17.6
C16:0	44.0	8.5	8.4
C18:0	4.5	2.4	2.5
C18:1	39.2	15.4	6.5
C18:2	10.1	2.4	1.5
C18:3	0.4	–	–
C20:0	0.1	0.1	–
Saturates	49.9	82.1	91.9
Monounsaturates	39.2	15.4	6.6
Polyunsaturates	10.5	2.4	1.5

MCFAs are found in the kernels of oil palm (*Elaeis guineensis*) and coconut palm (*Cocos nucifera*), of the *Arecaceae* palm family (Table 1) (6). They are also found in the milk of various animals, and C8:0, C10:0, and C12:0 are present in milk fat in the amounts of, respectively, 1.3–1.4, 2.7–3.3%, and 2.9–3.8 in cows; and 4.3, 12.8, and 6.6% in goats (7). Human breast milk also contains MCFAs, and their content has been reported to vary with the postpartum period (8, 9). C8:0, C10:0, and C12:0 are reported to contain 0.03–0.5, 0.3–0.6, and 2.0-3.1% of colostrum fat, 0.03–0.2, 0.5–1.6, and 3.1–6.8% of transition milk fat, and 0.03–0.2, 0.4–1.5, and 1.8–5.9% of mature milk fat, respectively (8, 9). It is thought that infants take in MCFAs from breast milk, and adults from dairy products (milk, butter, yogurt, etc.). According to the results of an epidemiological study of Japanese people, the average intake of MCFAs in adults is about 300 mg/day (10). It has been reported that MCFAs in breast milk are synthesized *de novo* from glucose in the human mammary gland, and secreted (11). In addition, it has recently been reported that most of the medium-chain fatty acids in breast milk exist as medium and long chain fatty acid oils containing both long and medium chain fatty acids in the same molecules (12, 13). This structure may also make sense.

MCFAs has been reported to enhance calcium and magnesium absorption (14) and improve fat and nitrogen absorption (15) in premature infants. In addition to these, MCFAs are known to be more easily digested and absorbed than LCFAs (16). Infants require large amounts of energy and nutrients for development, but because their digestive and absorptive capacities are not yet developed, therefore, it is very important to enhance the absorption of energy sources and nutrients for infant development. The mechanism by which MCFAs are provided directly by mothers through breast milk is interesting, considering the original physiological importance of MCFAs.

## Nutritional Characteristics of MCFAs

### Characteristics of Digestion and Absorption

Experiments with radioactive isotopes in 1951 showed that fatty acids less than C12:0 were rarely found in the thoracic duct lymphatics, but were mostly in the portal vein (17). It was later confirmed in animal experiments that there were no adverse effects, even when administered in large doses (3), and clinical studies on humans began. In the early 1960s, basic research was undertaken on the digestion and absorption in the intestinal tract of MCTs (18), their physiological effects, and their application to various diseases (19). In 1967 the symposium on MCTs was held at the University of Pennsylvania, and published in book form (2).

MCTs are digested very differently from long-chain triglycerides (LCTs); both are specifically degraded by tongue lipase, but MCTs are degraded 5–8 times faster and are also degraded in the stomach (16). According to recent studies, MCFAs-specific G protein-coupled receptor 84 (GPR84) is expressed on the tongue, and is reported to have some effect on taste *via* GPR84 (20).

The absorption pathway when ingesting MCTs and LCTs is also very different, with most of the MCTs and their degradation products being transported directly to the liver *via* the portal vein without forming bile micelles. The percentage transported *via* the portal vein is inversely correlated to the chain length of the fatty acid, with almost all C8:0 and about 60% of C12:0 migrating to the portal vein (21, 22). Therefore, MCTs composed of C8:0 and C10:0 do not form chylomicrons in the intestinal tract as LCTs do, and consequently do not increase postprandial blood triglycerides. Recent studies have revealed that some may be reconstituted into triglycerides in epithelial cells and transported into the bloodstream *via* lymphatic vessels, similar to LCTs (23).

In this way, direct intake of MCTs is unlikely to affect postprandial triglycerides, but since it supplies acetyl CoA to the liver, an increase in serum cholesterol may be seen if serum cholesterol is low due to malnutrition. This might be due to a temporary increase in cholesterol biosynthesis, since acetyl CoA is also the starting material for the biosynthesis of cholesterol. Under conditions such as fasting, starvation, and continuous intake of low-carbohydrate diet, lipolysis increases free fatty acids in the blood, and in addition, NAD+ increases in hepatocytes, which in turn increases ketone body synthase (17). On the other hand, due to the lack of pyruvate and alpha-ketoglutarate derived from glycolysis system by restricting carbohydrates, the reaction with acetyl CoA is reduced. As a result, fatty acid-derived acyl CoA is not sufficiently metabolized by the tricarboxylic acid (TCA) cycle and is allocated to ketone body production (17). When an individual ingests more MCTs than the TCA cycle and respiratory chain can process the acetyl CoA produced in the liver is converted to ketone bodies, causing an increase in blood ketone body concentration (17). The ketone bodies produced are transported to tissues other than the liver; for example, muscles, heart, kidneys, and brain, for utilization.

MCTs and LCTs also differ in their secretory stimulation of gastrointestinal hormones. In the stimulation of cholecystokinin (CCK) secretion, a gastrointestinal hormone related to the stimulation of pancreatic juice and bile secretion, MCTs have no effect on secretory stimulation (24). It is confirmed that, unlike LCTs, MCTs are not stored in the stomach, and migrate relatively quickly to the lower digestive tract. Thus, it can be inferred that coconut oil containing long-chain fatty acids (LCFAs) stimulates CCK secretion as well as LCTs in general. The fact that MCTs do not stimulate CCK secretion suggests that bile and pancreatic juice are not essential for the hydrolysis of MCTs. Furthermore, MCTs do not induce stimulation of CCK secretion, which seems to be related to their not causing the characteristic gastric distention and lying heavy of LCTs (25).

Acylation of MCFAs (C8:0 in many animals) is essential for the activation of ghrelin, which is secreted from the gastric wall during fasting. The physiological effects of ghrelin include stimulation of growth hormone and enhancement of food intake *via* the afferent vagal nerve. It has also been reported to increase gastric peristalsis, stimulate gastric acid secretion, and regulate insulin secretion. It is not clear whether the origin of C8:0, which is responsible for this ghrelin activation, is endogenous or exogenous. But it is known that MCTs, when ingested orally or intravenously, increase the blood concentration of acylated ghrelin in the fasting state (26, 27). In a study of anorexic adults, an increase in acylated ghrelin was confirmed by ingestion of 6 g of MCTs per day (27). In addition, it has been reported that in experiments using receptor knockout mice, MCFAs derived from ingested MCTs were stored in the cells of the stomach wall (28). This suggests that exogenous ingestion of MCTs may affect processes of appetite promotion and digestion.

MCTs and LCTs also differ in the stimulating of incretin secretion. There are two types of incretins, gastric inhibitory polypeptide (GIP) and glucagon-like peptide-1 (GLP-1), but the physiological roles of these two types differ. In addition to stimulating insulin secretion, GLP-1 has been reported to suppress appetite and promote fat metabolism *via* the central nervous system (29). MCTs, unlike LCTs, have been reported to act on GLP-1 but not on GIP (30). In clinical studies, MCTs ingested before meals were reported to induce a series of physiological changes, including lowered postprandial blood glucose levels, increased insulin secretion, suppression of food intake, and increased GLP-1 levels in the blood (31). MCTs and LCTs were also shown to differ in their effects on stimulation of gastrointestinal hormone secretion, such as ghrelin, CCK, and incretin.

### Endogenous MCFAs

Recent reports have shown a significant increase in endogenous MCFAs in the blood of healthy subjects with impaired glucose tolerance (32), long-duration endurance exercisers (33), and patients with recurrent coronary artery disease (34). The presence of MCFAs has also been reported in the exhaled air of certain cancer patients (35). Nowak et al. conducted an interventional study to discover blood biomarkers useful for detecting glucose intolerance, and to examine the underlying pathophysiological changes (32). They reported that the acylcarnitine ester concentration of MCFAs in the blood decreased over time to a greater extent during the oral glucose tolerance test in the group showing stronger insulin resistance. At this time, acylcarnitine, especially C10:0 and C12:0, was specifically detected, and there were no significant changes under C8:0 or over C14:0.

The presence of endogenous MCFAs has been reported not only in patients with diseases, but also in healthy individuals. During moderate-intensity exercise, the energy substrate in skeletal muscle becomes more dependent on fat instead of carbohydrate. Lehmann et al. (33) analyzed component changes in blood during moderate-intensity exercise and confirmed the presence of C8:0, C10:0, and C12:0 carnitine esters, respectively. They also confirmed that these are degradation products derived from C16:0. To evaluate their physiological significance, they confirmed that acylcarnitine in these MCFAs enhances fatty acid oxidation, from experiments on human myotube cells and mouse skeletal muscle. They stated that the detailed physiological significance is unknown, but is at least likely to be useful to us. Shi et al. reported a significant increase in MCFA (C8:0) in the blood during marathon running (36). As these studies suggest, many studies of MCTs have examined the effects of exogenous MCTs, but under certain physiological conditions, the carnitine esters of MCFAs and MCFAs may be produced endogenously and may have physiological effects.

## Various Clinical Application Studies

### Application to Gastrointestinal Malabsorption

MCTs have been used since the 1950s for energy supplementation in patients with lipid malabsorption, liver dysfunction, and other malnutrition conditions. The characteristics of MCTs make them easier to digest, absorb, and metabolize than LCTs. Malnutrition is associated with intestinal dysfunction, including increased intestinal permeability, malabsorption, and diarrhea. Therefore, malabsorption and increased intestinal permeability itself may hinder the effectiveness of nutritional support and recovery from malnutrition.

Because lipids are higher in calories than other nutrients, they are often added to foods to increase their energy density. But in severely malnourished children, who require the most additional dietary energy, lipid metabolism may be interfered with (37).

The use of MCTs as a fat source in protein-rich diets (up to 40–70%) has been reported to enhance lipid absorption in patients with malabsorption syndrome (17, 37–40).

When 40% of fat intake is replaced by MCTs, lipid absorption is enhanced by about 10%, compared to LCTs alone (41). In other words, replacing part of the lipid with MCTs seems to improve the absorption of LCTs. In addition, it has been reported that the absorption of Ca, Mg, and amino acids is increased in infants when MCTs are included in the diet (14, 17). Although there are some reports of clinical studies of MCTs, especially in premature infants, a review in the Cochran Library reported no clear effect of intake of MCTs on growth in children (42). Although there is some evidence of low certainty, the small number of trials and the small size of each trial population may be the reason for the lack of evidence showing a difference, and may indicate a need for more clinical studies.

A retrospective review was reported of clinical data on MCTs administered for 2 weeks as nutritional therapy to hospitalized patients, comparing parameters such as body weight, albumin, lipid levels, and uric acid before and after administration (43). During the 2 year study period, which began in January 2012, 46 of the 1,152 people admitted to the hospital for nutritional therapy were administered MCTs as cooking oil or enteral nutrition. Of those 46 patients, 21 had gastrointestinal dysfunction (improved in 15 patients), 15 had lymphatic abnormalities (improved in seven patients), five had dyslipidemia (improved in three patients), four had exocrine pancreatic insufficiency (improved in two patients), and one had epileptic seizures (no improvement). It was stated that administration of MCTs may be useful in the management of gastrointestinal malabsorption, pancreatic exocrine insufficiency, and dyslipidemia. However, it was noted that more randomized controlled trials with appropriate sample sizes and longer follow-ups were needed.

### Application to Epilepsy

MCTs have also been used as a ketone diet (modified Atkins diet), as a substitute for carbohydrates in patients with intractable pediatric epilepsy, a neurological disorder. Ketone diets, which are designed to produce high levels of ketone bodies, decrease seizure frequency in patients with refractory epilepsy. In the 1920s, several papers were published on the effects of fasting on epilepsy (44). Wilder et al. thought that if they could create a state of ketosis, they could achieve the same effect as fasting, so they implemented a dietary regimen and observed a dramatic reduction in epileptic seizures. The diet at this time is referred to as the classic ketone diet (45).

Later, Huttenlocher et al. developed a ketone diet (MCTs-ketone diet) that allows efficient ketone production with less lipids by using MCTs (46). Furthermore, the results of RCT studies have reported that the effects of the classical ketone diet and the MCTs-ketone diet are equivalent (47).

The Atkins diet is a low-carbohydrate diet developed by Dr. Atkins for weight loss in obese individuals. Based on this diet, a modified Atkins diet was developed for patients who cannot continue to consume the classic ketogenic diet (48).

The question of why such a ketone diet is effective for epileptic seizures has not yet been fully clarified. One of the hypotheses proposed is that acetoacetic acid, a type of ketone body, promotes the conversion of glutamate to glutamine, a cerebral neuroexcitatory transmitter, and then effectively converts glutamine to GABA, thereby suppressing neural excitation (49). It is also thought that a ketone diet may suppress the intracellular glycolytic system and reduce the ATP concentration in neurons, thereby inhibiting electrical neuronal membrane excitation (50).

In 2016, new findings showed that C10:0 inhibits neuronal excitation by binding to α-amino-3-hydroxy-5-methyl-4-isoxazolepropionic acid (AMPA) receptors at neuronal synapses (51). Other reports on the function of C10:0 in suppressing epilepsy (52, 53). There are *in vivo* data supporting this hypothesis that decanoic acid crosses the blood-brain barrier (54). This study suggests a role for C10:0 in the suppression of epileptic seizures through a ketogenic diet. A recent systematic review (SR) of 932 subjects (711 children aged 4 months to 18 years, and 221 adults aged 16 years and older) and 13 clinical studies on the efficacy of a ketogenic diet in drug-resistant epilepsy has been reported (55). In conclusion, all 13 studies were rated as high risk for detecting efficacy because they were not blinded and were conducted in small study populations. The SR stated that evidence for the use of a ketogenic diet, especially for adults, remains unclear, but did suggest that a ketogenic diet may have efficacy in children with drug-resistant epilepsy.

### Application to the Health Issues Among Elderly

Most of the clinical applications research of MCTs have been in the medical field, for post-surgical nutrition management and specific lipid absorption disorders. But in the 1980s, research started again on healthy subjects, in areas such as obesity prevention and sports activities. In the 2000s, studies have been particularly conducted on the elderly.

**Figure 2:**
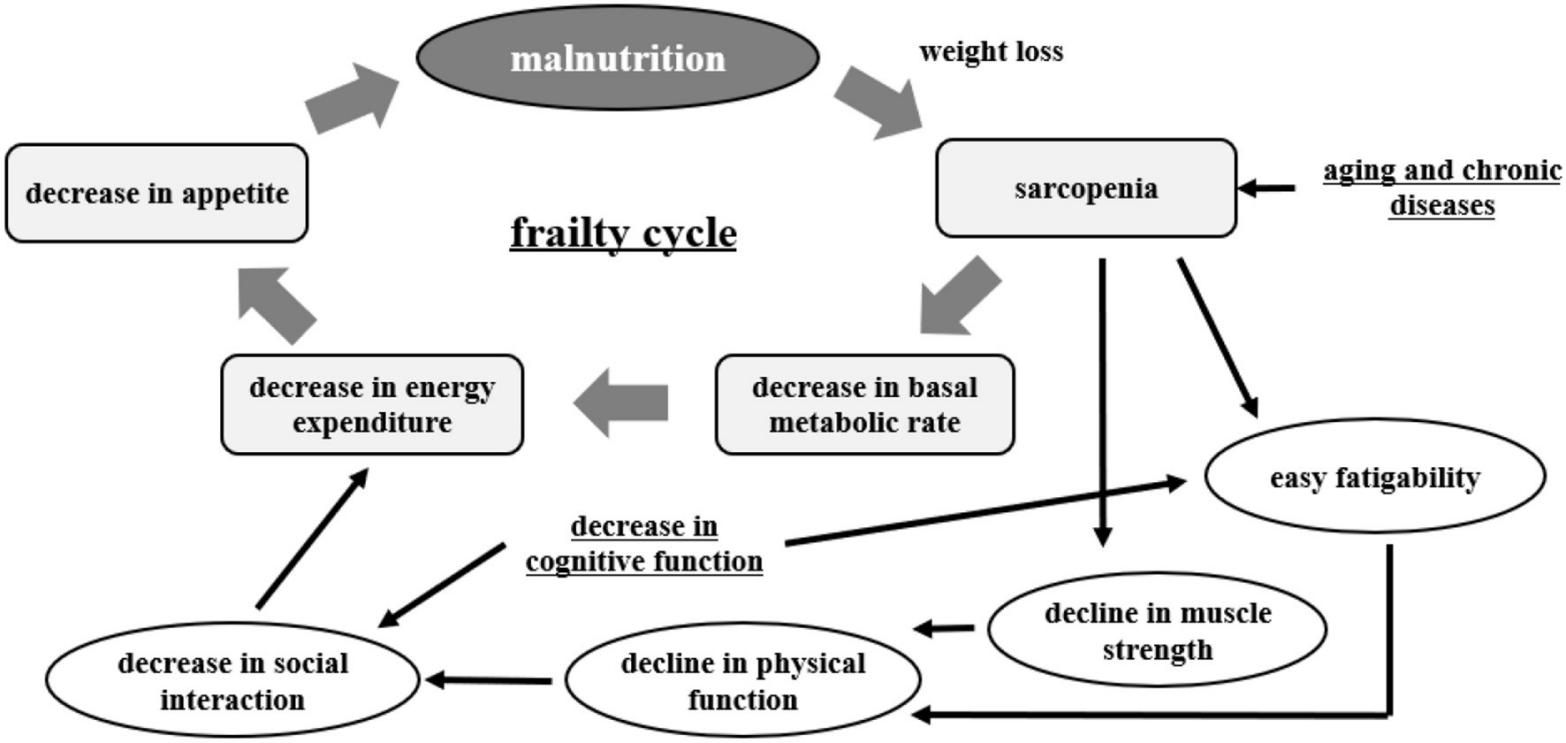
Frailty cycle [modified from Xue (57)].

Malnutrition, frailty, and dementia are major health issues among the elderly. A recent trilateral study from China, Japan, and Taiwan reported that the incidence of malnutrition is higher in people over 70 years old (56). Malnutrition is interrelated with sarcopenia, frailty, and the development of dementia. This is important in the matter of healthy life expectancy in the elderly (57) (Figure 2).

In the elderly, the balance of energy intake is considered to be negatively skewed by both a reduction in food intake resulting from complex factors (decreased activity, decreased appetite due to medication, decreased chewing and swallowing function, misperception, etc.) and increased energy metabolism due to chronic diseases and cancer.

Frailty is defined as “a condition in which mental and physical vitality declines with age, and life functions are impaired due to the coexistence of multiple chronic diseases, resulting in the emergence of mental and physical vulnerability, but with appropriate intervention and support, it is possible to maintain and improve life functions” (58).

Sarcopenia is a biological change in muscle mass and strength that occurs with aging. It is regarded as a core feature of frailty (59, 60). Sarcopenia is caused by a variety of changes associated with aging (61, 62). These include impaired neurological effects, chronic inflammatory conditions, oxidative stress, and decreased levels of sex hormones (60) and growth hormone (63). While these various factors are thought to be related, exercise and nutrition are expected to be effective in improving daily life. In particular, sarcopenia-obesity, which is a combination of those two conditions, is not only a combination of pathologies but is thought to have more metabolic abnormalities and muscle dysfunction, as well as a higher cardiovascular risk (59, 60).

The potential of MCTs in addressing the matters of malnutrition, frailty, and sarcopenia specific to the elderly is beginning to be studied. The aim of this approach is that, compared to LCTs, it is almost tasteless and odorless, so it can be easily ingested by adding it to the usual dishes for elderly people who eat a fixed meal menu, and it has less effect on blood lipids.

#### Application to Malnutrition

**Figure 3:**
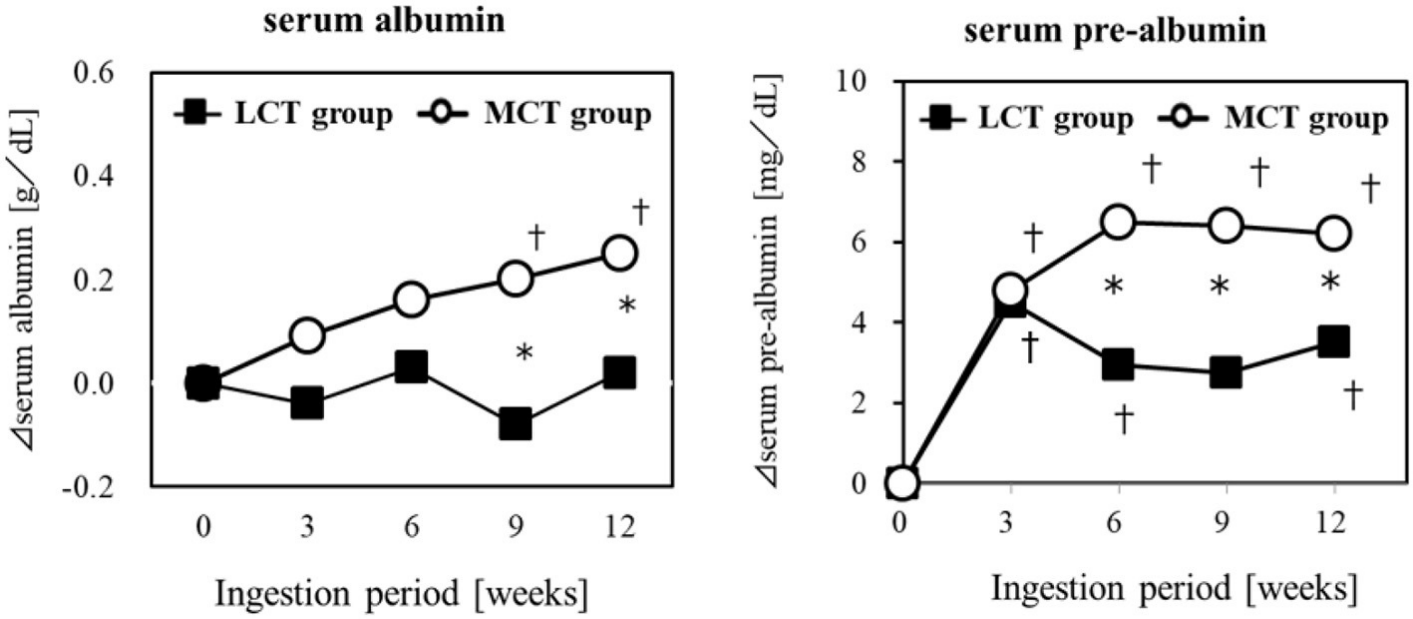
Improvement of malnutrition by MCTs ingestion. Six gram/day of MCTs or LCTs in liquid form, divided into 2 doses/day. Ingested for 12 weeks in older adults (24 subjects) at high risk for malnutrition. *Significant difference between the groups (*p* < 0.05). ^†^Significant difference from the start (*p* < 0.05).

Elderly residents of a geriatric care facility, not bedridden but at nutritional risk, were given a liquid test food containing 6 g of MCTs (C8:C10 = 75:25) or 6 g of LCTs, for 12 weeks, to evaluate the effect on their nutritional status. The results showed that Alb and pre-Alb in the blood changed over time in both groups, and pre-Alb and Alb were significantly higher in the MCTs group in the 9 and 12 weeks (64) (Figure 3). Weight, BMI, upper arm circumference, and lower leg circumference showed significant changes over time in the MCTs group but not in the LCTs group. Sebaceous thickness increased significantly over time in both groups. Blood creatine concentration, an indicator of skeletal muscle mass, did not increase in either group. In this study, the mechanism of the improvement of serum albumin by MCTs was described as the sparing effect of MCTs being rapidly metabolized as energy in the liver (65, 66), the increase of lipid-derived energy utilization by the activation of lipid oxidase in the liver (67), and the suppression of glycogenesis by the degradation of amino acids (66).

The authors concluded that 6 g of MCTs per day may significantly improve various blood parameters reflecting nutritional status, especially albumin, which is a risk indicator of malnutrition, compared to ingestion of LCTs in elderly people at risk of malnutrition. In studies that administered MCTs as central venous nutrition, improvement in nutritional status was confirmed without liver dysfunction (68, 69). The demonstration of the potential of MCTs in oral ingestion to improve malnutrition is significant, in that it can be expected to maintain and improve the health of the elderly through slight changes in their daily dietary habits.

#### Application to Frailty

**Figure 4:**
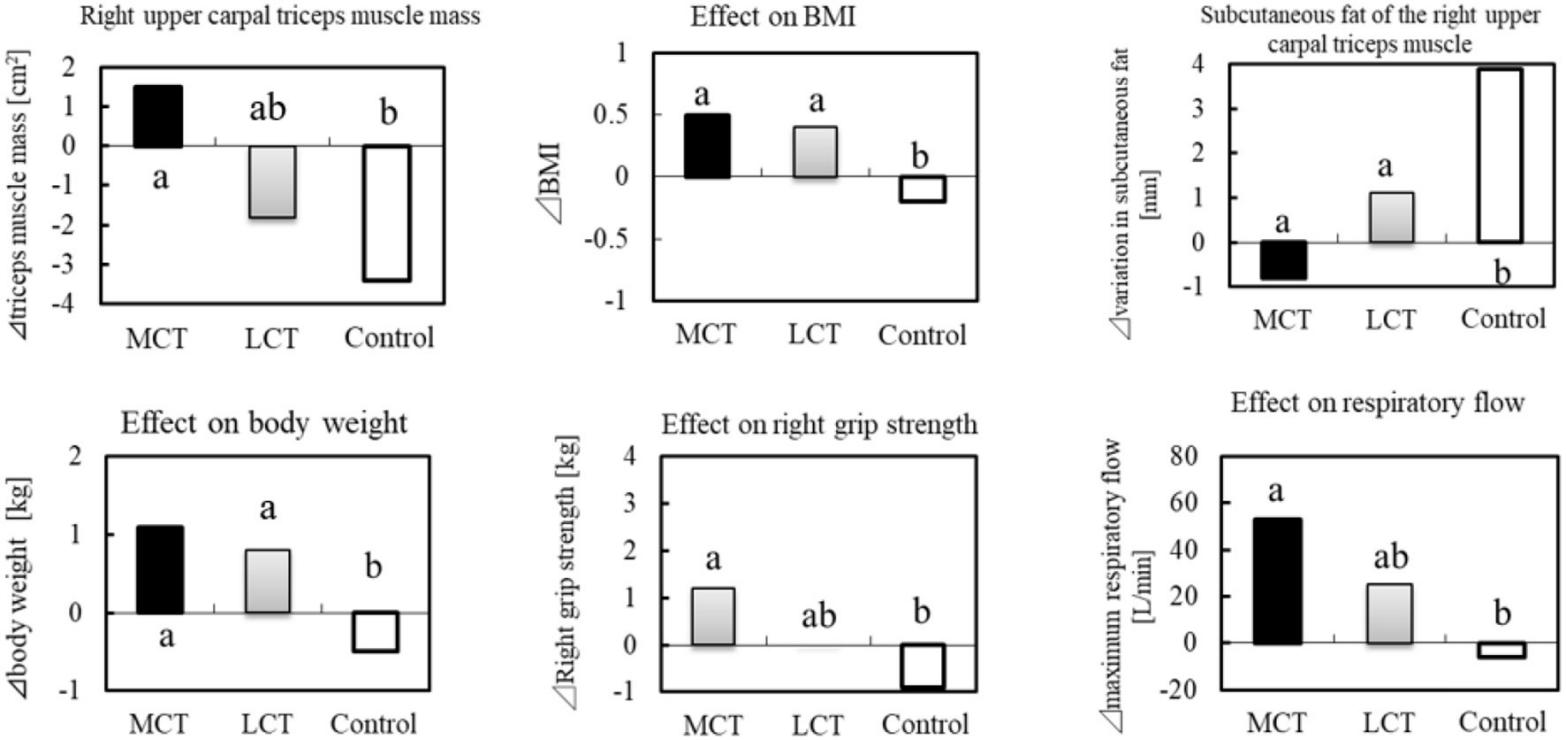
BMI, muscle mass and sarcopenia prevention in the elderly by MCTs ingestion. Thirty-six elderly residents were given MCTs at mealtime (6 g/day for 90 days). In addition, BCAA (1.2 g) and vitamin D (800IU) were commonly ingested in the MCT group, LCT group at the same time.

In a clinical study of frail elderly residents of a nursing home, the effects of 6 g of MCTs (C8:C10 = 74.9:25.1) on sarcopenia were examined (38 subjects, mean age 86.6 ± 4.8 years) during continuous ingestion for 3 months (70). In addition to the MCTs group, an LCTs group (rapeseed oil) and a control group (no oil or fat intake) were set up. The clinical study was conducted in three groups in a randomized, single-blind trial. In the study, 1.1 g of L-Leucine (Leu) and 20 μg of vitamin D (V.D) were ingested in combination in the LCTs and MCTs groups. The MCTs group reported significant improvement in right grip strength, 10-s leg opening/closing test, and maximum respiratory exhaust flow, compared to the control. In the 10-s leg opening/closing test, there was a significant difference from the LCTs group (Figure 4).

The same researchers later studied whether MCTs ingested alone had a similar effect on muscle strength and activities of daily living (ADLs) (71). In that study, 48 elderly residents of a nursing home (mean age 85.5 ± 6.8 years) ingested daily either 6 g of LCTs (LCTs group: rapeseed oil), 6 g of MCTs (MCTs group, C8:C10 = 74.9:25.1), or 6 g MCT + 1.2 g Leu + 20 μg V.D (LD + MCTs group). The MCTs group differed significantly from the LCTs group in performing a 10-second leg opening and closing test and a 30-second saliva swallowing test, and in their functional independence measure (FIM) score (a questionnaire for assessing ADL). But the authors noted that the limitations of their study were the small number of subjects and the possibility of bias due to the single-blind design. They also noted that since the study was conducted on frail, elderly Japanese, it is unclear whether this ingestion of MCTs would be effective for Westerners, or even for healthy elderly people. They also speculated that the mechanism for the effect of MCTs on muscle function is the activation of ghrelin by C8:0.

C8:0 is essential for the activation of ghrelin, which stimulates the release of growth hormone (72). Growth hormone and acyl ghrelin levels were significantly lower in the elderly than in young adults, suggesting that ingestion of MCTs may be more effective in the elderly (73). There was a positive correlation between the amount of MCTs administered and serum acyl ghrelin concentration, and it has been reported that 6 g/d of MCTs over a period of 2–6 weeks is necessary to increase acyl ghrelin in patients with anorexia nervosa (27). In cachexic patients with chronic respiratory disease, enteral administration of MCTs 3.0 g/d (C8:0 = 100%) for 2 weeks was shown to increase plasma acyl ghrelin concentrations. Preliminary studies conducted to determination dosage suggest that 6 g oil per day is well-tolerated in frail elderly people (28).

In support of the above mechanism, molecular mechanisms for the downstream effects of ghrelin acylation on protein metabolism have also been reported, including the activation of Akt/mTOR in the liver by ingestion of MCTs (74). Insulin and insulin-like growth factor (IGF-1) are typical mediators of Akt/mTOR, both of which have been reported to be increased by ingestion of MCTs (28, 31). In studies using animal models of chronic kidney disease, enhanced muscle protein metabolism, and mitochondrial function were reported (75). Thus, although MCTs are lipids, they may also affect the metabolism of albumin and muscle proteins.

Protein intake is generally recommended to improve malnutrition. But elderly people have resistance to protein intake, and tend to require more protein intake than younger people (76). In addition, a balanced intake of energy and protein is important when addressing malnutrition in the elderly. Recent studies have shown that high protein intake can increase skeletal muscle mass and muscle strength, but can also cause functional decline in endurance, mitochondrial function, and glucose tolerance (77). In order to prevent and address malnutrition, sarcopenia, and frailty, it may be useful to include MCTs, which are easily utilized as energy, along with an appropriate amount of protein intake, considering the specific physiological conditions of the elderly.

#### Application to Dementia

The social and economic impact of Alzheimer's disease (AD) is immeasurably huge, and it weighs heavily on humanity as we enter a society of longevity. The cause of the onset of AD is still unknown, but various studies suggest that lifestyle, along with aging, is a significant risk factor for developing AD.

One researcher has called AD “Type III diabetes” as it impairs glucose utilization by brain neurons (78). Glucose metabolism is reduced in the brain as cognitive function declines in healthy individuals, people with mild cognitive impairment (MCI), and AD patients (79). It has been reported that glucose metabolism is reduced by about 9% in the elderly, compared to the young, and by an additional 9% in MCI and AD, and by nearly 20% in dementia patients, compared to the young (79). On the other hand, ketone body metabolism has also been shown to be decreased in patients with MCI and AD (79). This suggests that it is difficult to supplement energy on one's own in cases of AD. When healthy elderly people consume a low-carbohydrate diet for a certain period of time, blood ketone bodies rise in parallel and are excreted in urine, but there is a report of a temporary improvement in cognitive function, correlated with the amount of ketone bodies excreted (80). Ketone bodies are an alternative energy source to glucose in the brain, and it has been found that ketone bodies are preferentially used rather than glucose (81). And recent studies have shown that the ability to metabolize ketone bodies is maintained to some extent in the AD brain (82). These studies suggest the possibility of using ketone bodies as an alternative energy source to improve cognitive function. It has been reported that there is a correlation between blood ketone levels and ketone body utilization in the brain (83). Since MCTs taken orally can increase blood ketone levels, the effects of MCTs on peripheral ketone levels and cognitive abilities of MCI and AD patients have been investigated in clinical studies.

Recently, a meta-analysis of clinical studies reported on ingestion of MCTs and cognitive function (84). In that meta-analysis, the search included literature up to March 1, 2019 (Table 2). The randomized controlled trial (RCT) meta-analysis reported that, compared to a placebo, ingestion of MCTs significantly elevated beta-hydroxybutyrate and showed trends toward improvement in cognitive function assessed by the Alzheimer's Disease Assessment Scale-Cognitive Subscale (ADAS-Cog) (84). The authors concluded that ingestion of MCTs may improve cognitive function in MCI and AD patients by inducing mild ketosis. The effects of ingestion of MCTs on the cognitive function of elderly people who are not AD patients, and younger people, have also been studied.

**Table 2:** List of reports on the effects of MCTs on cognitive function [Modified from Avgerinos et al. (84)].

**Study**	**Country**	**Study design**	**Number and type of patients**	**Diagnostic criteria for inclusion**	**Study groups (composition of MCTs)**	**Intervention dose**	**Duration of intervention**	**Plasma BHB measurement method**	**Cognitive measure**
Fortier (85)	Canada	RCT	52 with MCI	Subjective memory complaint, MoCA, MMSE	MCTs vs. Placebo (C8:0 = 60%, C10:0 = 40%)	30 g/d	6 months	Colorimetric assay using an automated clinical chemistry analyzer (Dade Behring Inc., Newark, US)	MMSE, MoCA, trail making test, Stroop test, verbal fluency, digit symbol substitute ion, Boston naming test
Ota Part A (86)	Japan	RCT	20 with mild/moderate AD	NINCDS-ADRDA	MCTs vs. Placebo (C8:0 = 30.3%, C10:0 = 9.8%, C12:0 = NR)	20 g/d	2 days	Enzymatic method (SRL Corp., Tokyo, Japan)	WAIS III, WMS-R, Stroop test, trail making test
Chan (87)	Malaysia	RCT	41 with mild/moderate/severe AD	MMSE	Coconut oil vs. Placebo (NR)	60 ml/d	6 months	NA	MMSE, clock drawing test
Rebello (88)	USA	RCT	6 with MCI	National Institute on Aging	MCTs vs. Placebo (NR)	56 g/d	6 months	NR	ADAS-Cog, trail making test digit symbol test
Yang (89)	Spain	RCT	44 with AD	Institutionalized AD patients (unclear diagnostic criteria)	Coconut oil vs. placebo (NR)	40 ml/d	3 weeks	NA	MMSE (Spanish version)
Henderson (90)	USA	RCT	152 with mild/moderate AD	NINCDS-ADRDA and DSM-IV criterial	MCTs vs. Placebo (C8:0 = 100%)	20 g/d	3 months	BHB Liquicolor diagnostic kit (Stanbio Laboratory, L.P., Boerne, US)	MMSE, ADAS-Cog
	USA	RCT	20 with probable AD or amnestic MCI	NINCDS-ADRDA criteria	MCTs vs. Placebo (C8:0 = 100%)	40 ml/d	2 days	Enzymatically, using procedure 310-UV (Sigma Diagnostics Inc., Livonia, US)	MMSE, ADAS-Cog, Stroop test, paragraph recall
Ota Part B (86)	Japan	1 arm trial	19 with mild/moderate AD	NINCDS-ADRDA criteria	MCTs (C8:0 = 30.3%, C10:0 = 9.8%, C12:0 = NR)	20 g/d	3 months	Enzymatic method (SRL Corp., Tokyo, Japan)	WAIS III, WMS-R, Stroop test, trail making test
	USA	1 arm trial	10 with very mild/mild/moderate AD	National Institute on Aging	MCTs + low carb/ high fat diet (NR)	22.5–45 ml/d	3 months	NR	MMSE ADAS-cog
Ohnuma (91)	Japan	1 arm trial	20 with moderate/severe AD	NINCDS-ADRDA	MCTs (NR)	20 g/d	3 months	ELISA (KAINOS Laboratories Inc., Tokyo, Japan).	MMSE, ADAS-cog
	USA	Case report	55 with probable mild/moderate AD	MMSE	MCTs (C8:0 = 100%)	20 g/d	18.8 ± 9.2 months	NA	MMSE
Newport (92)	USA	Case report	Young onset sporadic AD	Clinical diagnosis, MMSE scores, MRI, APOE4 carriage	MCTs + coconut oil (NR)	165 ml/d	2.5 months	Precision Xtra Glucose and Ketone Monitoring System (Abbott Laboratories., Chicago, US)	MMSE, ADAS-cog
Farah (93)	USA	Case report	Probable AD	MMSE, MoCA, FDG PET	MCTs (C8:0 = 100%)	20 g/d	~3 months	NA	MMSE, MoCA

*AD, Alzheimer's Disease; ADAS-Cog, Alzheimer's Dis. Assessment Scale-cognitive subscale; BHB, beta-hydroxybutyrate; CONTAB, Cambridge Neuropsychological Test Automated Battery; C-SSRS, Columbia Suicide Severity Rating Scale; C8, octanoic acid; C10, decanoic acid; FDG PET, fluorodeoxyglucose (18F) positron emission tomography; MCI, Mild Cognitive Impairment; MCTs, Medium Chain Triglycerides; MMSE, Mini Mental State Examination; MoCA, Montreal Cognitive Assessment Scale; NA, Not Applicable; NINCDS-ADRDA, National Institute of Neurological and Communicative Disease and Stroke and the Alzheimer's Disease and Related Disorder Association; NM scale, Nishimura geriatric rating scale; NR, Not Reported; WAIS, Wechsler Adult Intelligence Scale; WMS-R, Wechsler Memory Scale-Revised*.

O'Neill et al. conducted a randomized, double-blind crossover study of 80 subjects aged 55–80 years with MCI (94). They reported that when 30 g/day of MCTs (C8:C10 = 55:45) were ingested for 2 weeks, there was no significant difference in performance in cognitive function tests such as short-term memory, attention, and executive function from the comparison control, although there was a significant increase in beta hydroxybutyrate concentration. They noted that there was no significant difference in cognitive function in this study due to factors such as significantly elevated blood ketone levels after ingestion, which were lower than in previous studies, and the short intervention period.

Abe et al. conducted a randomized, double-blind, parallel-group study of 36 elderly people aged 65 years and older, without diagnoses of AD, who were admitted to a nursing home (95). The intervention consisted of three groups: a control group (no oil added); an LD-MCT group [1.2 g Leu, 20 μg V.D, 6 g MCTs (C8:C10 = 75:25)]; and an LD-LCT group [Leu, V.D + 6 g LCTs (canola oil)]. Each oil was administered at dinner time, for 3 months. The MMSE scores of the LD-MCT group were significantly higher than those of the control group and significantly improved compared to the pre-intervention group, but the LD-LCT group showed no difference from the control.

Further, Abe et al. conducted an intervention trial by assigning 64 elderly people with a mean MMSE score of 17.1 to three groups and having them intake LCT, or LD-MCT, or MCT only, at 6 g with dinner for 3 months (95). The MMSE scores decreased by 0.7 points in the LCT group. But they increased by 3.4 points in the LD-MCT group and by 3.5 points in the MCT group, both significant increases compared to the LCT group. However, in their discussion, the authors pointed out the limitations of their study, including the small number of subjects, and the single-blind design.

Ashton et al. conducted a randomized, parallel-group study in which 30 healthy young adults in their twenties were assigned to three groups (96). The results of the ingestion of MCTs (C8:C10 = 30:70) of 0 g (control), 12, and 18 g daily for 8 weeks showed that performance in a trail-making test and a correct and reverse recitation of numbers were significantly improved after 2 or 3 weeks by ingestion of MCTs.

Otsuka et al. studied the effect of dietary habits on cognitive decline, using epidemiological methods (10). The eight-year follow-up study on dietary habits and risk of cognitive decline was conducted among community-aged men and women with MMSE scores of 28 or higher at the start of the study. The group with MMSE scores of 27 or lower after follow-up was defined as the cognitive decline group, and the group with MMSE scores of 28 or higher was defined as the equal/maintained group. In analysis, when the risk of cognitive decline associated with an increase of one standard deviation (+1 SD) in fatty acid intake was calculated, the odds ratios were significantly lower at 0.855 and 0.840 for a 1 SD increase in intake of short-chain fatty acids (SCFAs) and MCFAs. The daily intake of MCFAs in the target population was 302.3 ± 231.9 mg (mean ± SD), and although a 2 SD increase was calculated by the estimated formula to reduce the risk of MMSE score decline by 8 and 17%, the actual comparison of the target population showed declines of 0 and 13.7%, which did not necessarily agree with the estimated formula. This study suggests that dietary intake of MCFAs in daily life may be involved in maintaining cognitive function.

Ingested MCTs are digested and absorbed, and most are transported *via* the portal vein to the liver, where they are metabolized and raise blood ketone concentrations, but a small portion is transferred to the peripheral blood as MCFAs and esters (97, 98). Many studies suggest that ketone bodies, an alternative energy source, are involved as a factor for MCTs to affect AD, but it is also possible that MCFAs, not ketone bodies, directly affect neurological functions. It has been suggested that non-polar lipids of low molecular weight cross the brain barrier, and that MCFAs transferred to the peripheral blood may be metabolized to ketone bodies by astrocytes in the brain and utilized, although this was a cellular-level study (99). It has also been reported that MCFAs increase neuronal mitochondrial biosynthesis, leading to increased brain glucose metabolism (100). In addition, the maintenance and improvement of brain function involves the promotion of neuronal regeneration and maturation, and C10:0 and C12:0 may act as agonists for these processes (101).

Abe et al. described the involvement of hippocampal synapses in the acylation of ghrelin as a factor in the observed effects of relatively low doses of MCTs on cognitive function (102). These findings suggest that the effects of MCTs on cognitive function may be compounded by the effects of MCFAs and ghrelin acylation, as well as ketone bodies. Since AD is irreversible, we hope that more clinical studies will be conducted on MCI and other diseases in the future. Since the amount of MCTs that are effective in improving cognitive function at this stage is relatively high, we hope that improvements will be investigated in this regard as well.

### Application to Exercise and Physical Activity

Energy substrates of skeletal muscle during physical activity include carbohydrates and lipids. Carbohydrates are more efficient in producing adenosine triphosphate (ATP) (103, 104) and carbohydrates are used more frequently (105). In recent years, various studies have been conducted on the utilization of lipids in exercise.

It is known that more energy is required during exercise than at rest, and that energy expenditure per unit of time increases in proportion to the intensity of exercise (106). Among athletes who exercise heavily, some experience an imbalance between energy intake and energy expenditure. In 2007 the American College of Sports Medicine published a position stand on “the female athlete triad,” and listed “low energy availability with or without an eating disorder” instead of “disordered eating” as one of the three main characteristics. One of its statements includes: “the first aim of treatment for any triad component is to increase energy availability by increasing energy intake and/or reducing exercise energy expenditure” (107). From this perspective, the intake of lipids is considered important as an efficient energy source in athletes with a high level of physical activity.

Carbohydrates, along with fats, are used as energy substrates, but most carbohydrates in the body are stored as glycogen, mainly in the muscles and liver. During exercise, glycogen stored in muscles is metabolized and used as an energy substrate for muscles. It is known that the amount of glycogen stored in the body is much less than the amount of fats stored as adipose tissue or ectopic fat, but depletion of glycogen in muscle reportedly results in poor exercise quality and muscle fatigue (108). It is important to know how to recover muscle glycogen quickly, in order to prevent deterioration in the quality of daily training. The recovery of muscle glycogen requires the intake of sufficient amounts of carbohydrate as a raw material (109), but it has long been known that the recovery of muscle glycogen is accelerated by the intake of protein in addition to carbohydrate. Protein intake promotes the secretion of the incretins GIP and GLP-1, which increases insulin secretion and enhances glucose uptake into muscle (110).

It is known that GIP and GLP-1 secretion is also stimulated after lipids intake, and GIP secretion is strongly stimulated after intake of LCTs (24). In contrast, animal studies have shown that GIP is secreted only in small amounts after intake of MCTs, and GLP-1 secretion is promoted (111). Terada et al. showed in animal experiments that intake of milk-derived lipids together with carbohydrate after running exercise significantly promoted muscle glycogen recovery, compared to carbohydrate intake alone (112). A similar study in humans showed that ingesting lipids in addition to carbohydrates after exercise may promote an increase in blood insulin levels and a decrease in blood glucose levels (112). These studies suggest that carbohydrates plus lipids are more effective than carbohydrates alone in terms of glycogen recovery after training.

Since then, studies of lipids and exercise have shifted in perspective from energy supplementation to increased lipid metabolism in energy metabolism during exercise. The significance of lipid intake in exercise is that by increasing the ratio of lipid to energy intake, lipid metabolism is enhanced and aerobic respiratory capacity is increased (113). In a human study, Zajak et al. reported that continuous ingestion of a high-fat diet increased the lactate threshold and maximal oxygen uptake (VO_2_max) and enhanced lipid metabolism during exercise (114). In recent years, the effects of high-fat diets on athletes have also been studied. It has been reported that lipid metabolism during exercise is enhanced in athletes, which may improve their endurance exercise capacity (115, 116). The mechanism of lipid metabolism enhancement by high-fat diets is reported to be the increase of mitochondria in skeletal muscle and activation of fatty acid degrading enzymes in mitochondria *via* peroxisome proliferator-activated receptor delta (PPARδ), which is a ligand for fatty acids (117–119).

However, human studies of the effects of high-fat diets on endurance exercise performance have not been uniformly positive, and in some cases have been reported to affect performance adversely (120). This may be due to the increase in body fat and weight caused by the continuous intake of high-fat diets and suppression of the glycolytic system (112). It has also been reported that continuous intake of high-fat diets increases the expression of PDH kinase-4 (PDK-4) (121), which negatively regulates the activity of pyruvate dehydrogenase (PDH), an important enzyme in the glycolytic system, and suppresses the glycolytic system (122, 123). Since carbohydrate utilization as a percentage of energy substrate increases in proportion to exercise intensity, suppression of the glycolytic system decreases anaerobic exercise capacity (103). Therefore, continuous intake of high-fat diets may adversely affect performance in some types and forms of exercise.

Some of the adverse aspects of LCT-based high-fat diets on exercise quality could be partially improved by replacing them with MCTs. Several studies have been conducted. Mumme et al. conducted a meta-analysis using 13 human studies and reported that replacing a portion of ingested LCTs with MCTs reduced body fat and weight (124). Fukazawa et al. reported that high-fat diets based on MCTs enhanced lipid metabolism without increasing PDK-4 expression in skeletal muscle in animal experiments, indicating that it may be possible to enhance lipid metabolism without suppressing the glycolytic system (125). In addition, it has been reported that the enhancement of lipid metabolism by MCTs and the associated improvement in endurance exercise capacity may be stronger than that achieved by LCTs (126–128). Nosaka et al. conducted two studies in which humans were given 6 g of MCTs per day for 2 weeks. In both tests, 40 min of moderate-intensity exercise followed by high-intensity exercise to exhaustion was performed, one with LCTs as a control (129) and the other with carbohydrates as a control (130). Both studies showed that the duration of high-intensity exercise to exhaustion was significantly prolonged in the MCTs ingestion group (129, 130).

On the other hand, some reports have obtained results showing no improvement in performance with ingestion of MCTs. In studies of the effect on performance of single MCTs ingested immediately before exercise, MCTs did not enhance fat oxidation during exercise (131–133). In addition, it has been reported that diarrhea occurred in subjects when ingesting high amounts of MCTs (133). Jeukendrup et al. studied in several ways the effects of ingestion of MCTs on performance during exercise (134–136). None of the studies showed any benefit in ingesting MCTs, compared to ingestion of carbohydrates during exercise. Rather, they reported that gastrointestinal complaints occurred and adversely affected performance (136). In addition, studies of continuous ingestion of high-dose MCTs have shown no improvement in performance, compared to ingestion of LCTs (137, 138). These results suggest that it is unlikely that a single ingestion or ingestion during exercise will provide efficacy. Furthermore, attention should be paid to dosage and administration, as ingesting a large amount of MCTs at one time may cause gastrointestinal complaints. The studies that showed performance benefits were all studies in which most subjects were female and were given relatively low doses of MCTs continuously (Table 3). Although there may be gender differences in the effects on performance of ingesting MCTs, there are still many issues to be examined in the future.

**Table 3:** Effect of MCTs on performance during exercise.

**Ingestion period**	**Subjects**	**Amount of ingestion**	**Composition of MCTs**	**Control**	**Exercise intensity, time**	**Performance**	**Reference**
Just before exercise	10 men	30 (g)	C8:0 = 96%	LCTs	70 (%VO_2_max), 1 (h)	No difference	(131)
(single ingestion)	12 men	25 (g)	NR	CHO	60 (%VO_2_max), 1 (h)	No difference	(132)
	6 men	25 (g)	C8:0 = 100%	water	65 (%VO_2_max), 2 (h)	No difference	(133)
During exercise	8 men	29 (g)	C8:0 = 100%	CHO	57 (%VO_2_max), 3 (h)	No difference	(134)
	8 men	26.6 (g)	C8:0 = 99%	CHO	57 (%VO_2_max), 1.5 (h)	No difference	(135)
	7 men	85 (g)	C8:0 = 99%	CHO	60 (%VO_2_max), 2 (h)	Negative effect (gastrointestinal complaints)	(136)
continuous ingestion	7 men	34 (g, 7 days)	NR	LCTs	80 (%VO_2_max), until exhaustion	No difference	(137)
	12 men	60 (g, 14 days)	C <8:0 ≤6% C8:0 = 67% C10:0 = 23% C>10:0 ≤4%	LCTs	75 (%VO_2_max), until exhaustion	No difference	(138)
	1 man, 7 women	6 (g, 14 days)	C8:0 = 74% C10:0 = 26%	LCTs	80 (%VO_2_max), until exhaustion	Improved	(129)
	8 women	6 (g, 14 days)	NR	CHO	70 (%VO_2_max), until exhaustion	Improved	(130)

*CHO, Carbohydrate; LCTs, Long-Chain Triglyceride; NR, Not reported*.

Exercise is undertaken not only to improve performance, but also to maintain and improve health. In recent years, attention has been paid to the importance of physical activity in daily life, the relationship between non-exercise activity thermogenesis (NEAT) and obesity (139), and the relationship between the amount of physical activity in daily life, life expectancy, and disease risk (140, 141). The ability to adjust the ratio of carbohydrate and fat utilization as energy substrates in response to external stimuli such as exercise and physical activity in daily life is indicated by the index of metabolic flexibility (142, 143), which has been reported to be strongly correlated with the development of obesity and Type II diabetes (144–146). Enhancing fat metabolism during exercise and daily physical activity is considered to be important for the maintenance and promotion of good health. Nosaka et al. conducted a study in which sedentary middle-aged and elderly people were given 6 g of MCTs per day for 2 weeks, to compare their lipid metabolic performance with that of carbohydrates during low-intensity exercise at the daily activity level. The results showed that continuous ingestion of 6 g/d of MCTs enhanced lipid metabolism, compared to carbohydrates (147, 148). Recently, Tsujino et al. conducted a study in which sedentary obese people were given 2 g/d of MCTs for 2 weeks. They reported that compared to ingestion of LCTs, ingestion of MCTs enhanced fat oxidation during low-intensity physical activity (149).

Fat metabolism may also be enhanced by daily endurance exercise (150, 151) and daily physical activity (152). In animal studies, ingestion of MCTs and exercise have been shown to additively enhance fat metabolism and reduce visceral fat (153). Recent changes in our living environment (e.g., widespread use of automobiles and increased time spent at home due to the pandemic) have resulted in an unintended decrease in our physical activity. We would like people not only to ingest MCTs, but to combine them with exercise and physical activity in their daily lives.

### Application to Prevention of Obesity

Obesity caused by overnutrition and inactivity is a potential health issue, and a cause of subsequent development of lifestyle-related diseases. Fat accumulation occurs in insulin-sensitive tissues such as liver, adipose tissue, muscle, and heart. Fat accumulation in these tissue cells inhibits the metabolism of fatty acids and glucose, and induces various health dysfunctions *via* cytokines.

Many clinical studies have been conducted on the prevention of obesity by ingestion of MCTs. Since 2010 there have been several clinical research articles published on the effects of ingestion of MCTs on body composition, weight loss, and energy expenditure (124, 154–157). Among them are two SRs (124, 158). The review by Mumme et al. selected and analyzed randomized clinical studies with a duration of ingestion of at least 3 weeks, comparing MCTs (consisting of C8:0 and C10:0) with LCTs for body composition, weight, serum lipids, and other endpoints in healthy men and women (124). Thirteen clinical trials (*n* = 749) were selected (Table 4). It was found that compared to LCTs, MCTs resulted in a significant reduction in body weight, waist circumference, hip circumference, total body fat, total subcutaneous fa, and visceral fat. The discussion in this review noted that many of the individual clinical studies lacked sufficient information for a complete quality assessment, and that commercial bias was detected. Although there was no heterogeneity, it was noted that the study design differed with respect to duration of ingestion, dosage, and method of controlling energy consumption. In conclusion, it was stated that replacing LCTs in the diet with MCTs may cause a modest reduction in body weight and body composition without adversely affecting lipid profiles.

**Table 4:** List of reports on the effects of MCFAs ingestion on body weight, body composition, and blood lipids [modified from Mumme and Stonehouse (124)].

**References**	**Methods, duration**	**Subjects**	**Country**	**Intervention (composition of MCTs)**	**Control**	**Outcomes**
Yost et al. (159)	DB P, 4–12 weeks	16 obese women, 29−44 years	US	800 kcal/d *via* formula, energy from MCTs (~21 g/d) (C8:0 = 58.3%, C10:0 = 21.9%, C12:0 = 0.3%)	LCTs	BW
Temme et al. (160)	SB P, 6 weeks	60 adults, BMI 20–30	Netherland	Margarine and foods, 10% energy from MCTs (~24 g/d) (C6:0 = 33.3%, C8:0 = 25.3%, C10:0 = 41.5%, C12:0 = 0.2%)	LCTs	BW, TG, TC, HDL-cholesterol, LDL-cholesterol
Feldheim et al. (161)	C–O, 2 × 4 weeks	35 women, 19–24 years, normal BMI	Czech Republic	Fat provided, 5% energy from MCTs (~12.5 g/d) (NR)	LCTs	BW
Krotkiewski et al. (162)	DB P, 4 weeks	66 obese, perimenopausal women	Sweden	579 kcal/d, 13% energy from MCTs (9 g/d) (NR)	LCTs	BW, BC, TG, TC
Matsuo et al. (163)	P, 12 weeks	13 men, 18–20 years, normal BMI	Japan	Liquid formula supplement, 20g MLCTs (C8:0 = 7.3%, C10:0 = 2.4%, C12:0 = NR)	LCTs	BW, total adipose, TG, TC, HDL-cholesterol, LDL-cholesterol
Tsuji et al. (164)	DB P, 12 weeks	78 adults	Japan	Breakfast bread containing ~4% energy from MCTs (10 g/d) (C8:0 = 74.4%, C10:0 = 25.6%, C12:0 = 0.0%)	LCTs	BW, BC, TG, TC
Kasai et al. (165)	DB P, 12 weeks	82 adults, mean BMI = 25	Japan	Breakfast bread containing 14 g MLCTs (C8:0 = 9.7%, C10:0 = 3.3%, C12:0 = NR)	LCTs	BW, BC, TG, TC, HDL-cholesterol, LDL-cholesterol
Nosaka et al. (166)	DB P, 12 weeks	64 adults, mean BMI = 25	Japan	14 g margarine containing MCTs (5 g/d) (C8:0 = 39.0%, C10:0 = 13.0%, C12:0 = NR)	LCTs	BW, BC, TG, TC
Bourque et al. (167)	C–O, 2 × 27 days	17 obese women, mean age 44 years, mean BMI = 32	Canada	Fat mixture containing 20% energy from MCTs (~54 g/d) (C8:0 = 19.4%, C10:0 = 23.6%, C12:0 = 3.9%)	LCTs	BW, BC, TG, TC, HDL-cholesterol, LDL-cholesterol
St Onge et al. (168)	C–O, 2 × 4 weeks	25 overweight men, mean age 43 years	Canada	Structured oil containing 20% energy from coconut oil (C6:0 = 0.2%, C8:0 = 37.0%, C10:0 = 30.4%, C12:0 = 3.6%)	LCTs	BW, BC, blood lipid levels
Roynette et al. (169)	SB C–O, 2 × 6 weeks	32 overweight men, 18–45 years	Canada	Structured oil containing 13% energy from coconut oil 13% (48 g/d) (NR)	LCTs	BW, BC
St Onge et al. (170)	DB P, 16 weeks	49 overweight adults, 19–50 years	US	12% energy from MCTs, women: 18 g/d; men: 24 g/d (C8:0 = 55%, C10:0 = 44%, C12:0 = NR)	LCTs	BW, BC, TG, TC, HDL-cholesterol, LDL-cholesterol
Xue et al. (171)	DB P, 8 weeks	101 adults, BMI >22	China	25-30 g/d MLCTs oil (C8:0 = 9.7%, C10:0 =3.3%, C12:0 = NR)	LCTs	BW, BC, TG, TC, HDL-cholesterol, LDL-cholesterol

*BC, Body composition; BMI, Body mass index; BW, Body weight; CHO, Carbohydrate; C–O, Crossover; DB, Double-Blind; HDL, High-Density lipoprotein; LCTs, Long-Chain triglycerides; LDL, Low-Density lipoprotein; MCTs, Medium-Chain triglycerides; MLCTs, Medium long-chain triglycerides; NR, Not reported; P, Parallel; SB, Single-Blind; TC, Total cholesterol; TG, Triglycerides*.

Bueno et al. conducted a meta-analysis of randomized controlled trials to determine whether individuals assigned to replace at least 5 g of diet-derived LCTs with MCTs for at least 4 weeks would show positive changes in body composition (157). Three of the studies (158, 172, 173) included in this review by Bueno et al. (157) were not included in the above review by Mumme and Stonehouse (124). In an overall analysis including all studies, individuals who replaced dietary LCTs with MCTs underwent significant changes in body weight, body fat, and waist circumference. They concluded that although the results were statistically significant, the evidence available is not of the highest quality, and any recommendation to replace dietary LCTs with MCTs should be made with caution.

Reviewing the above two reports, it seems possible that MCTs have beneficial effects on body composition, body weight, and energy metabolism (174), but further examination is required. In particular, if an experiment will measure weight changes in the range of several 100 g per month, it will be necessary to measure body weight and fat mass in suitable facilities and under appropriate conditions, and it must be conducted under appropriate nutritional management for obese people.

Many studies have reported on the inhibitory effect of ingestion of MCTs on fat accumulation. Total daily energy expenditure is generally composed of three components: basal metabolic rate (about 60%), diet-induced thermogenesis (DIT, about 10%), and physical activity (about 30%). The basal metabolic rate depends on body size and does not vary greatly for an individual. Therefore, an increase in energy expenditure resulting from ingestion of MCTs may have an effect on DIT, or physical activity, or both. An SR on the effect of ingestion of MCTs on DIT showed that MCTs significantly increased DIT, compared to LCTs (175). The effects on substrate metabolism during physical activity have also been recently reported, and ingestion of MCTs has been shown to enhance fat oxidation in sedentary subjects (147, 149).

Several molecular mechanisms have been reported to promote lipid oxidation by ingestion of MCTs, suggesting the existence of the following mechanisms to enhance the oxidation of stored fat (176).

I. increase in noradrenaline secretion after ingestion of MCTs suggests a temporary promotion of lipid oxidation in adipose and muscle tissues through activation of the sympathetic nervous system and long-term activation of intracellular mitochondrial metabolism (177–180).
II. MCFAs may directly enter cells and activate receptors such as PPARs in the nucleus, thereby activating mitochondrial metabolism and enhancing lipid and energy metabolism (181, 182).
III. Activation of GLP-1 by MCTs and its non-reactivity with GIP inhibit postprandial fat synthesis and promote postprandial lipid oxidation (111).
IV. As an indirect effect, MCTs may increase satiety and reduce excessive food intake by activating GLP-1 in the daily diet (29, 31, 172).

At relatively high doses of MCTs ingested, the ketone bodies produced may stimulate lipolysis by increasing LPL activity in blood vessels (119, 183).

## New Clinical Application Research

It is well-known that bacterial components derived from intestinal bacteria induce pro-inflammatory cytokines such as interleukins through macrophages and dendritic cells. Studies of intestinal immunity to ingested exogenous polyunsaturated fatty acids have been reported (184), and several studies on intestinal immunity to MCTs (MCFAs) have been reported recently (185–188). Among them, some are negative about the effects of MCFAs on immunity (185, 186) and some are positive (187, 188).

Studies of infection-preventive and anti-inflammatory effects of MCTs against pathogenic *E. coli* in piglets have been reported (187). De Keyser et al. (187) confirmed the antimicrobial activity of MCFAs *in vitro*, when added to *E. coli* plates and incubated at varying pH values. Next, lipopolysaccharide was administered intravenously to piglets raised with MCTs. After its administration, the height of villi and the depth of the crypt consistently decreased in both the antibiotic and MCT groups. Subsequently, there was recovery of villi in the MCT group but not in the antibiotic group. The MCT group was also reported to have higher serum Immunoglobulin A (IgA) levels and more IgA-positive plasma cells and goblet cells in the jejunum than the antibiotic group. The study concluded that in the post-weaning period, the possible antimicrobial effect of MCFAs on E. coli and the protective effect on the small intestine were superior to antibiotics, and that the results had an effect on growth promotion in piglets.

In experiments with rats, lipopolysaccharide was administered in a similar manner, and the responses were compared (188). In the group that was administered MCTs for about 10 days previously, IgA secretion was increased compared to the control group that was administered saline and corn oil. It was reported that the expression of IL-6, which affects IgA secretion, was confirmed by mRNA and was increased in the MCT group, while the increased expression of pro-inflammatory cytokines and chemokines in the ileum was greatly suppressed by ingestion of MCTs. The mRNA expression of the Th2 IgA-stimulated cytokine IL-10 in the ileum and Bayer plates was significantly higher in the MCT group than in the control group. In contrast, the mRNA expression of interferon-γ, which is a cytokine that inhibits IgA secretion in Th1, was suppressed by MCTs. These results suggest that ingestion of MCTs modulates the immune response to lipopolysaccharide and enhances the expression of secretory IgA.

Although further research is needed to elucidate the effects of MCFAs on intestinal immunity and their mechanism of action, one hypothesis is that the effects of MCFAs may be related to the effects of royal jelly on the immune system (189). Royal jelly contains 10-hydroxydecanoic acid, which is structurally similar to decanoic acid. 10-hydroxydecanoic acid has been reported to promote the induction of differentiation of M cells, which are important for the production of IgA antibodies in small intestinal epithelial cells (189). As another mechanism, GPR84 is expressed in the intestine and especially in the cecum, and it has been reported that GPR84 reacts with C9:0 to C12:0 fatty acids as an agonist and activates macrophages (190). It has been reported that the cecum, which was thought to be useless in human beings, plays an important role in the regulation of IgA secretion in the intestine in general (191). Therefore, MCTs containing C10:0 or C12:0 may be involved in the activation of macrophages and the regulation of IgA secretion *via* GPR84 in the cecum.

Along with immune response, another aspect of homeostasis in living organisms is resistance to oxidative stress. Ketone bodies with reducing power have been reported to have various physiological functions (192), among which are physiological functions related to oxidative stress, such as anti-inflammatory and antioxidant effects. However, since a significant increase in blood ketone concentration was observed when more than 7 g of MCTs were ingested (193), it is unlikely that the antioxidant effect of MCTs can be expected without deliberate ingestion of MCTs in normal adults. Recently, it has been reported that MCFAs may directly enhance antioxidant capacity in neuroblastoma cell experiments (194). This effect is at least partially based on the enhancement of catalase activity, independent of changes in catalase gene expression. It also decreased the level of oxidative stress in the cells and reduced the cell death induced by H_2_O_2_. The concentration of the ketone body, β-hydroxybutyrate, had no effect on this process. Oxidative stress is considered to be an etiological factor in several neurodegenerative diseases, such as AD, and oral intake of MCTs has demonstrated beneficial effects in improving cognitive function. The factor of this improved cognitive function is generally considered to be that the ketone bodies resulting from the metabolism of MCFAs act as a glucose-replacement energy source in the brain. These results suggest that MCFAs may be involved in maintaining neuronal function by directly reducing oxidative stress levels, independent of ketone bodies.

The clinical application of MCTs in cancer patients has been studied (195, 196). MCT-rich diets (ketone diets) are being studied in combination with standard therapies such as chemotherapy and radiation therapy in advanced stage IV cancer or brain tumors that are difficult to remove surgically. For a variety of reasons, it is difficult to conduct high-quality clinical research on cancer patients, but more research is expected in the future.

It has been reported that MCFAs can improve clinical symptoms in patients with fatty acid oxidation disorders, especially in cardiomyopathy, a condition in which the heart is in a state of energy deficiency (197). It has been reported that patients with myocardial infarction associated with type II diabetes have a poor prognosis and ischemia/reperfusion injury, and such myocardial tissue has been reported to have decreased UCP3 metabolism (198). Genetic modification of UCP3(+/–) mice with MCT has been shown to inhibit reactive oxygen species (ROS) production and improve cardiac function (198). Related to cardiac disease, there is a rare and incurable disease, triglyceride deposit cardiomyovasculopathy (TGCV), which causes a specific large accumulation of triglycerides in the myocardium and coronary arteries, resulting in severe congestive heart failure (199). This disease was first reported in heart transplant patients. There is a primary type in which the gene for adipocyte triglyceride lipase (ATGL) is partially mutated, and an idiopathic type that is highly associated with diabetes (199). MCT (tridecanoate) has been shown to be useful in ATGL knockout mice and has potential for clinical application in TGCV patients (199). This study may have useful implications for dyslipidemia associated with diabetes mellitus. Once again, it makes us think about the usefulness and importance of MCTs in treating lifestyle-related diseases.

## Conclusion

**Figure 5:**
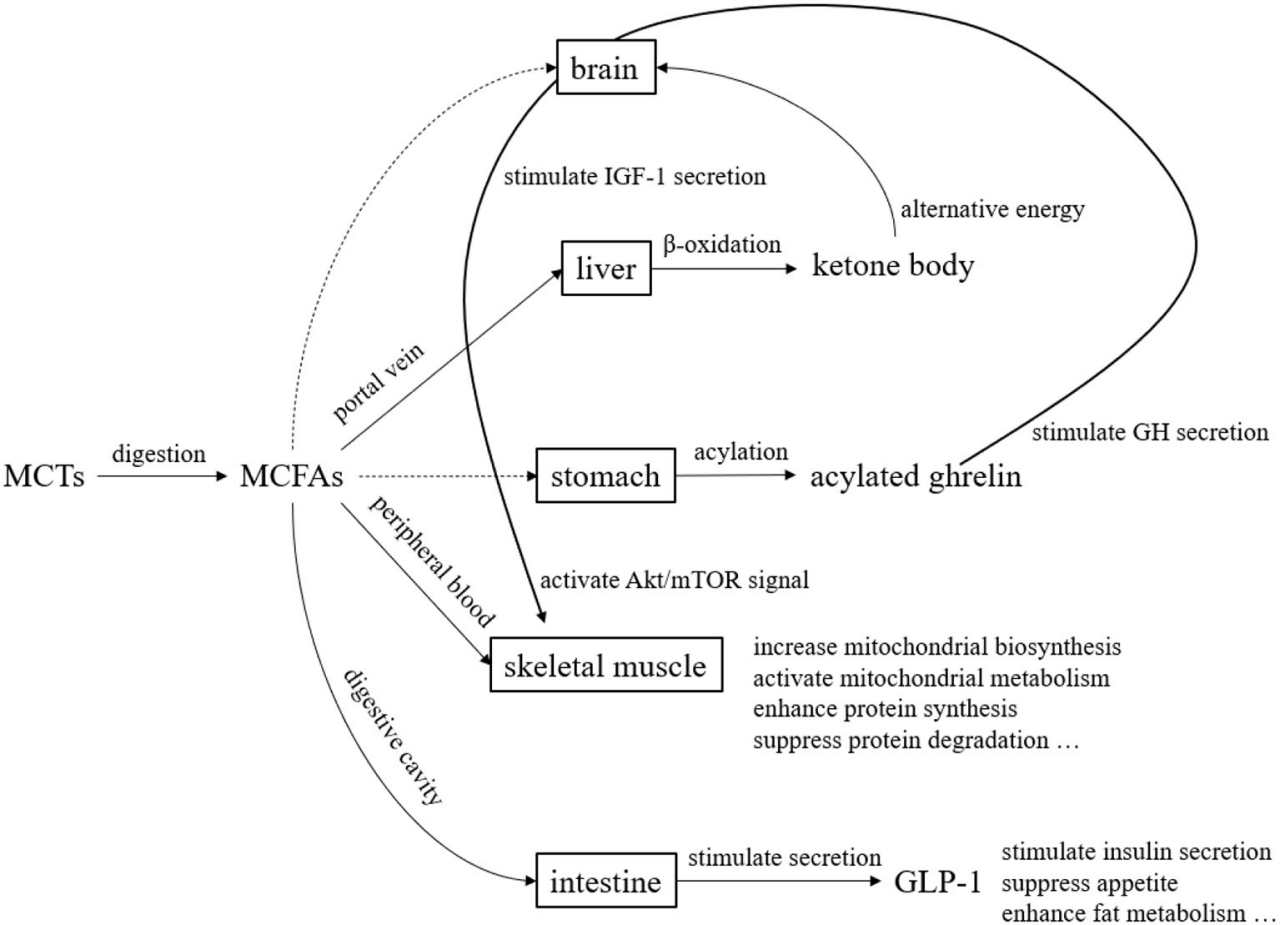
Overview of metabolism and functional expression of MCTs. MCTs are mostly transported to the liver *via* the portal vein after digestion and absorption. Since MCTs do not require carnitine for transfer to the mitochondria, they are quickly beta-oxidized and become energy. During this process, ketone bodies are produced, which serve as an alternative energy source to glucose in the brain. Although the pathway is unknown, acylated ghrelin levels in the blood increase after MCTs ingestion. Acylated ghrelin enhance the secretion of growth hormone (GH), which in turn stimulates the secretion of insulin-like growth factor 1 (IGF-1). Furthermore, IGF-1 enhance protein synthesis and suppress protein degradation by activating Akt/mTOR signaling. MCFAs transferred to the peripheral blood act on skeletal muscle to enhance mitochondrial biosynthesis and mitochondrial metabolic activity. MCFAs also stimulates the secretion of glucagon-like peptide 1 (GLP-1) from small intestinal L cells.

MCTs have been studied since the 1950s and used in the medical field and other fields since the 1960s. They are now widely used in food and non-food fields. In the past, they were used in the medical field mainly for the purpose of efficient energy supply. In recent years, their use has been studied not only in the medical field but also in healthy people, as a source of ketone bodies and for their physiological functions (Figure 5). Over the past several decades, MCTs have been the subject of clinical application studies in a variety of areas. However, the fatty acid composition of MCTs used in those clinical studies may not always be the optimal composition ratio. This may be because the MCTs used in the studies were commercially available MCTs, and the physiological effects of individual fatty acids and ketone bodies were not previously understood. The results of various clinical studies and basic research on individual medium-chain fatty acids and ketone bodies summarize their physiological significance (Table 5). In the future, we hope that clinical application studies using MCTs designed with a focus on the physiological functions of individual MCFAs will be promoted. MCFAs are biosynthesized in the mammary gland of the mother, and are present in small amounts in breast milk and dairy products. This makes them a lipid component that we intake throughout our lives, from infancy to adulthood. Although the physiological significance is unknown, it is known that under certain physiological conditions, endogenous MCFAs are present. Therefore, we believe that MCTs are lipids with great potential in nutritional physiology. We hope that through future research and development, they will become still more useful in maintaining and improving our daily health.

**Table 5:** Differences in each physiological function by chain length of MCFAs.

**Physiological function**	**More effective medium-chain fatty acid species**	**Examples of expected clinical applications**
Acylation of ghrelin	C8:0 >> C10:0	Application to malnutrition, sarcopenia
Production of ketone body	C8:0 > C10:0	Application to epilepsy, dementia
Enhancement of mitochondrial metabolism	C10:0 > C8:0	Application to physical activity
Enhancement of GLP-1 secretion	C10:0 >> C8:0	Application to obesity, hyperglycemia

## Author Contributions

Both authors listed have made a substantial, direct, and intellectual contribution to the work and approved it for publication.

## Conflict of Interest

SW and ST are employed by the Nisshin OilliO Group, Ltd., Japan.

## Publisher's Note

All claims expressed in this article are solely those of the authors and do not necessarily represent those of their affiliated organizations, or those of the publisher, the editors and the reviewers. Any product that may be evaluated in this article, or claim that may be made by its manufacturer, is not guaranteed or endorsed by the publisher.
